# Supplementary material for: Structural basis for ubiquitylation by HOIL-1
Source: Front Mol Biosci. 2023 Jan 6;9:1098144. doi: 10.3389/fmolb.2022.1098144 (PMC9853177; doi:10.3389/fmolb.2022.1098144)
Supplement: Supplementary file 1 [file DataSheet1.PDF]

frontiers

Supplementary Material

1 Supplementary Figures and Tables

1.1 Supplementary Figures

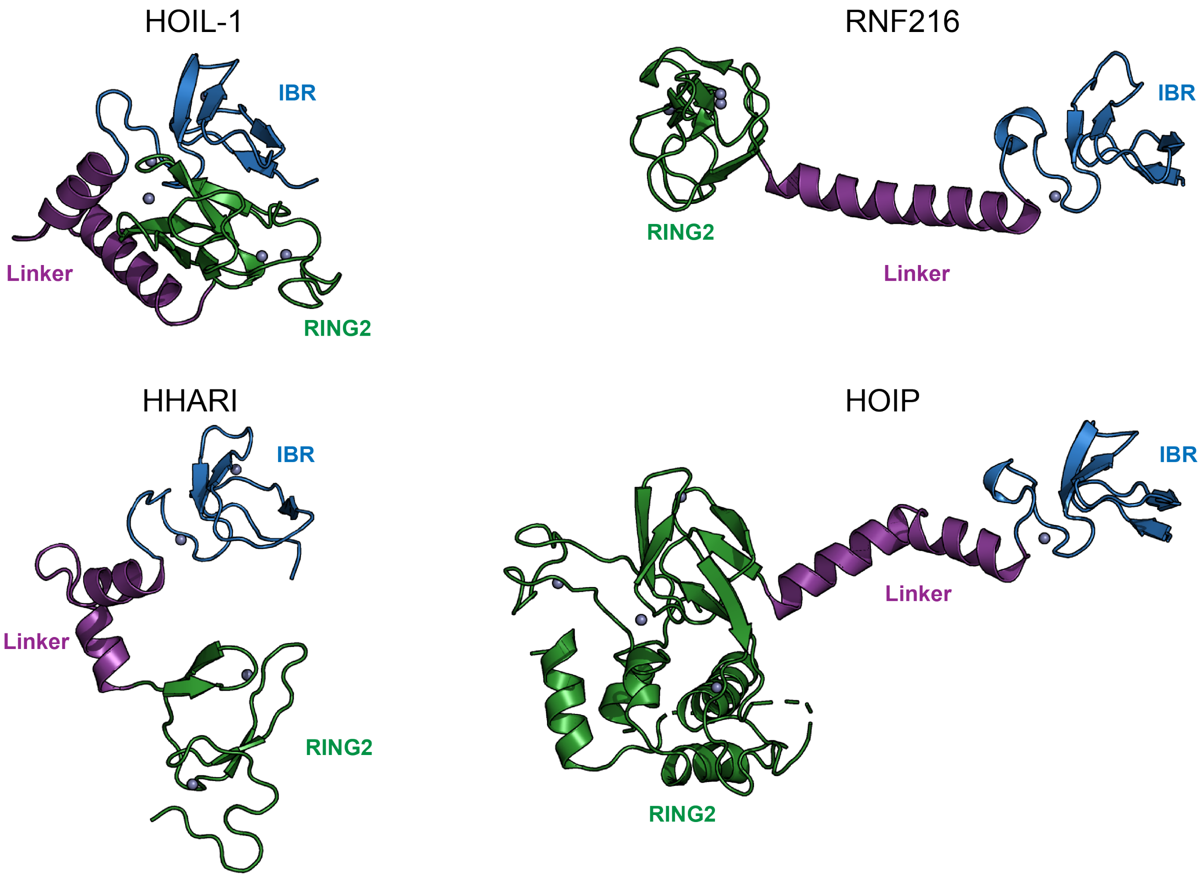

Supplementary Figure 1. Comparison of IBR-RING2 orientations in RBR ligases. The structure of HOIL-1 is compared with the IBR-RING2 conformations of the activated ligases RNF216 (PDB: 7M4M), HHARI (PDB: 7B5L) and HOIP (PDB: 5EDV). Structures are shown as ribbon models with Zn^{2+} ions as grey spheres.

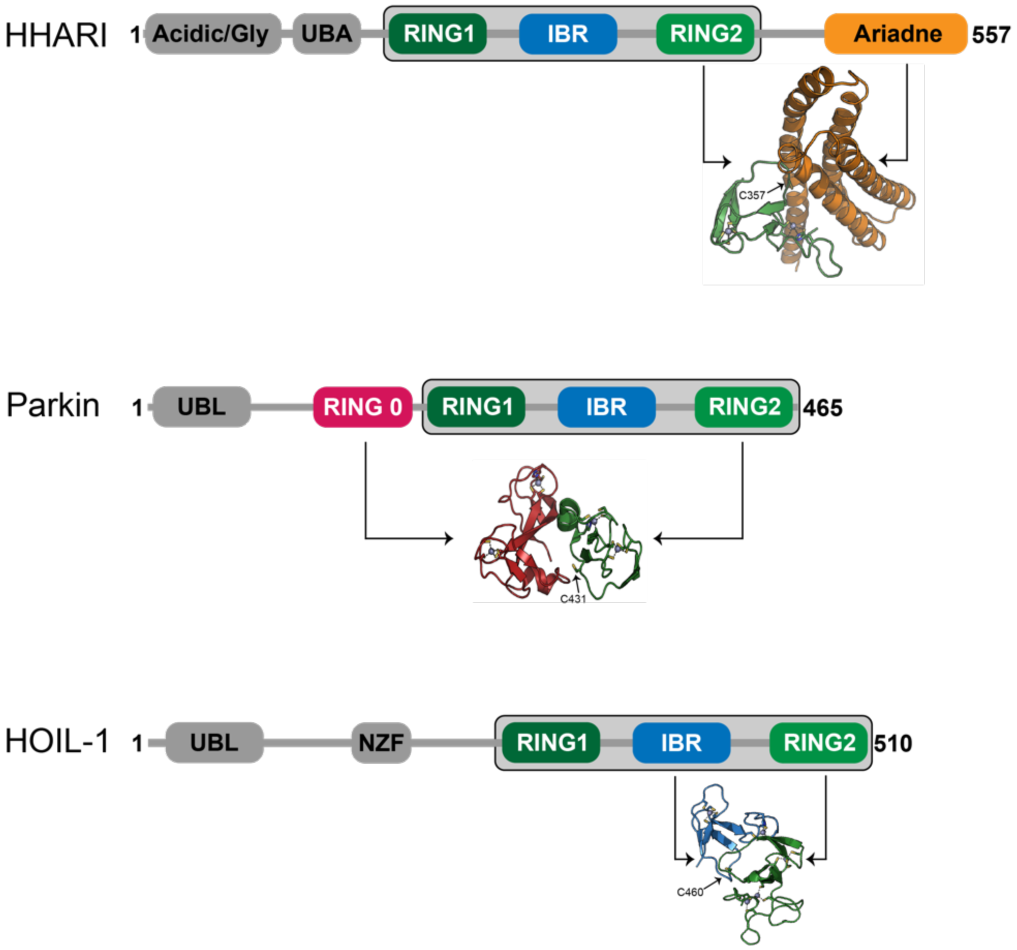

Supplementary Figure 2. Domain architecture and structural comparison of intramolecular RING2 interactions of HHARI, Parkin and HOIL-1. The RBR core module is indicated by a grey box. (Domain acronyms: UBL = Ubiquitin Like domain; NZF = Npl4 Zinc Finger; UBA = Ubiquitin Associated domain; Acidic/Gly = Acidic and Glycine rich domain; Ariadne = Ariadne domain; RING0/1/2 = Really Interesting New Gene domain; IBR = In Between RING domain) The intramolecular complexes between RING2 and corresponding domains are shown as a ribbon model. The Zn^{2+} coordinating residues and catalytic cysteines of HOIL-1, HHARI (PDB: 4KBL) and Parkin (PDB: 5C1Z) are indicated. Zn^{2+} ions are shown as grey spheres.

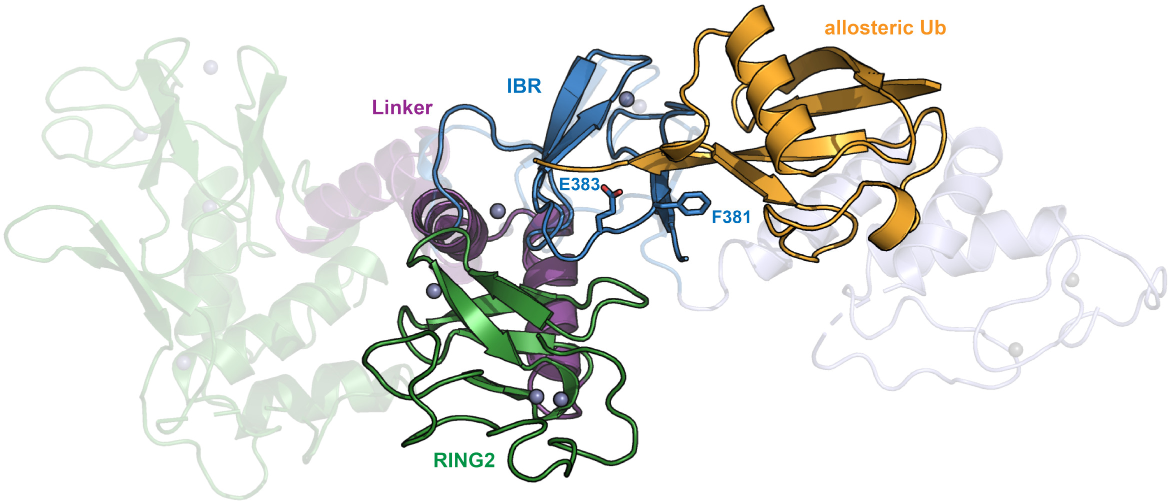

Supplementary Figure 3. Structural overlay of HOIL-1 IBR-RING2 with the active conformation of HOIP (PDB: 5EDV; translucent ribbon model) in complex with ubiquitin (orange). The structures are superimposed over the entire length of the IBR domains. Residues F381 and E386 are essential for HOIL activation (indicated as ball-and stick model).

1.2 Supplementary Table

Table S1: Data collection and refinement statistics

**Data collection**	
Space group	P 1 2_1 1
Cell dimensions	
a, b, c (Å)	53.91, 59.25, 57.21
α, β, γ (°)	90.00, 90.31, 90.00
Wavelength (Å)	1.2829
Resolution range (Å)	41.16 - (2.32 - 2.24)
Total reflections	33358 (3291)
Unique reflections	16782 (1651)
Completeness (%)	95.79 (95.81)
$I / \sigma I$	10.14 (3.75)
R_{merge}	0.08046 (0.2176)
$CC_{1/2}$	0.989 (0.914)
**Refinement**	
Resolution range (Å)	41.16 - 2.24
Reflections	16771
R_{work} [a]/ R_{free} [b]	24.73/29.45
Number of atoms	2342
Protein	2254
Zn^{2+}	10
Water	78
B-factors average (Å^2)	40.41
Protein (Å^2)	40.77
Zn^{2+} (Å^2)	26.16
Water (Å^2)	31.57
R.m.s. deviations	
Bond lengths (Å)	0.008
Bond angles (°)	1.00
Ramachandran	
favoured, allowed, outliers (%)	93.71, 4.90, 1.40

Statistics for the highest-resolution shell are shown in parentheses.

[a] $R_{work} = \sum_{hkl} ||F_{obs}(hkl)|-|F_{calc}(hkl)||/\sum_{hkl}|F_{obs}(hkl)|$.

[b] R_{free}= the cross-validation R factor for 5% of reflections against which the model was not refined.
